# High-Resolution and Quantitative X-Ray Phase-Contrast Tomography for Mouse Brain Research

**DOI:** 10.1155/2015/530580

**Published:** 2015-10-20

**Authors:** Yan Xi, Xiaojie Lin, Falei Yuan, Guo-Yuan Yang, Jun Zhao

**Affiliations:** ^1^School of Biomedical Engineering and Med-X Research Institute, Shanghai Jiao Tong University, Shanghai 200030, China; ^2^Department of Neurology, Ruijin Hospital, School of Medicine, Shanghai Jiao Tong University, Shanghai 200030, China

## Abstract

Imaging techniques for visualizing cerebral vasculature and distinguishing functional areas are essential and critical to the study of various brain diseases. In this paper, with the X-ray phase-contrast imaging technique, we proposed an experiment scheme for the *ex vivo* mouse brain study, achieving both high spatial resolution and improved soft-tissue contrast. This scheme includes two steps: sample preparation and volume reconstruction. In the first step, we use heparinized saline to displace the blood inside cerebral vessels and then replace it with air making air-filled mouse brain. After sample preparation, X-ray phase-contrast tomography is performed to collect the data for volume reconstruction. Here, we adopt a phase-retrieval combined filtered backprojection method to reconstruct its three-dimensional structure and redesigned the reconstruction kernel. To evaluate its performance, we carried out experiments at Shanghai Synchrotron Radiation Facility. The results show that the air-tissue structured cerebral vasculatures are highly visible with propagation-based phase-contrast imaging and can be clearly resolved in reconstructed cross-images. Besides, functional areas, such as the corpus callosum, corpus striatum, and nuclei, are also clearly resolved. The proposed method is comparable with hematoxylin and eosin staining method but represents the studied mouse brain in three dimensions, offering a potential powerful tool for the research of brain disorders.

## 1. Introduction

Animal modeling is an important method for the study of brain disease, such as intracranial aneurysms [1] and cerebral ischemia [2]. To understand the occurrence, pathogenesis, and development of brain disorders, it is critical to explore the microvascular network and differentiate its functional areas. Traditional 3D imaging methods for brain research mainly include X-ray computed tomography (CT) and magnetic resonance imaging (MRI) [3]. However, both methods have limitations, primarily the poor soft-tissue contrast of X-ray CT and poor spatial resolution of MRI.

Recently, X-ray phase-contrast imaging (PCI) has gained special attention owing to its soft-tissue imaging capability [4, 5]. The PCI technique is based on phase shifts of incident X-rays caused by the imaged object instead of its absorption properties, which are commonly used in traditional X-ray imaging methods. According to the X-ray imaging theory and experimental results, the PCI technique is superior in imaging low-density materials, such as soft tissues [6]. Many imaging methods for PCI have been proposed representing the sample using X-ray phase information [6]. Among them, the propagation-based PCI (P-PCI) method has the simplest system configuration [7] and is widely used in synchrotron radiation stations and X-ray laboratories.

However, in practical applications, due to the nonignorable X-ray source size and scattering inside the sample, the P-PCI method is limited in imaging small samples (several centimeters) and simple structures. To improve its imaging efficiency, we have proposed air-filled microbubbles as the contrast medium, and the results show it is promising in biomedical applications [8, 9]. In this paper, we extend the microbubble-based P-PCI method, using air-tissue interfaces to generate large phase shifts to facilitate the two- and three-dimensional imaging of mouse brain. The mouse brain is prepared following our proposed method filling air into its vascular network. Having the high spatial resolution by P-PCI method and enhanced contrast at air-tissue interface, the network of blood vessels of the air-filled mouse brain is highly visible in our experiments. However, in P-PCI tomography, the high-contrast edges introduce artifacts to the reconstructed images with conventional filtered backprojection (FBP) method, in which low-contrast areas are drowned in noise. To address this problem, we employ phase-retrieval (PR) method and redesign the reconstruction kernel to reduce undesired noise and maintain its spatial resolution.

The rest of this paper is organized as follows. In Section 2, we describe the PR-FBP method and give the new designed reconstruction kernel. The preparation scheme of air-filled brain is presented in detail. In Section 3, we compare the imaging results between conventional X-ray imaging and P-PCI to show the superior performance of P-PCI method and present the three-dimensional reconstruction results with PR-FBP algorithm. Finally, Section 4 gives the conclusion of our work.

## 2. Materials and Methods

### 2.1. Principle of P-PCI

The P-PCI method is based on the phase shift of X-ray instead of its change in amplitude. Considering the case that a monochromic and coherent X-ray beam illuminates the object with wavelength *λ*, the object can be characterized by its complex refractive index. Here, *β* is the absorption index and *δ* denotes the refractive decrement of the object. The transmittance function of the object can be described as

(1) $$\begin{array}{c} T\left(\;x,z\;\right)=\exp\left(\;-\frac{1}{2}\mu \left(\;x,z\;\right)+i\varphi \left(\;x,z\;\right)\;\right), \end{array}$$

where *μ*(*x*, *z*) and *φ*(*x*, *z*) are the amplitude change and phase shift of the incident X-rays, respectively, which are the integrals of the absorption and refractive index of object along the ray path *y*, defined as

(2) μx,z=4πλ∫βx,y,zdy,φx,z=−2πλ∫δx,y,zdy.

Passing through the object and propagating a short distance, X-rays arrive at the detector plane and form a Fresnel diffraction pattern, expressed as [10]

(3) $$\begin{array}{c} I_{D}\left(\;x,z\;\right)={\left|\;T\left(\;x,z\;\right)*P_{D}\left(\;x,z\;\right)\;\right|}^{2}, \end{array}$$

where *D* is the distance between the sample and the detector and *P*_*D*_ is the Fresnel propagator, defined as

(4) $$\begin{array}{c} P_{D}\left(\;x,z\;\right)=\frac{1}{i\lambda D}\exp\left(\;\frac{i\pi \left(x^{2}+z^{2}\right)}{\lambda D}\;\right). \end{array}$$

The image formation function (3) can be simplified with the transport of intensity equation, which restricts a short propagation distance [11], written as

(5) $$\begin{array}{c} I_{D}\left(\;x,z\;\right)=I_{0}\left(\;x,z\;\right)-\frac{\lambda D}{2\pi }\nabla \left[\;I_{0}\left(\;x,z\;\right)\nabla \varphi \left(\;x,z\;\right)\;\right], \end{array}$$

where *I*_0_(*x*, *z*) is the pure absorption image.

**Figure 1:**
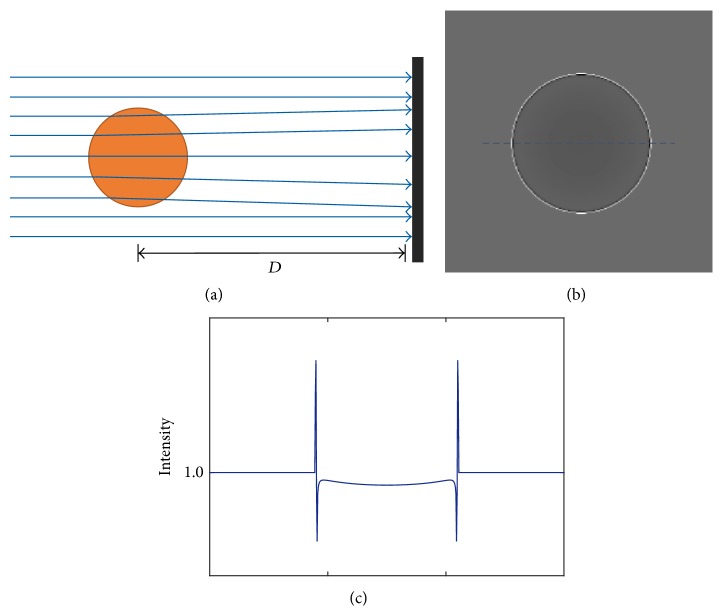
Illumination of P-PCI. The incident X-rays are refracted at structural boundaries (a), which results in bright-and-dark fringes in the captured image (b). (c) plots the intensity profile of the dashed line in (b).

An intuitive explanation of P-PCI is that the propagation directions of the incident X-rays are refracted at the interface of two different materials as shown in Figure 1, and consequently bright-and-dark fringes are formed in the recorded images (Figure 1(b)). This is the so-called edge-enhanced effect. In our previous studies [8, 9], the X-ray bending effect was used to focus incident X-rays into a bright point in detector plane by air-filled microbubbles. As its extension, in this paper, we prepare air-filled mouse brain and image it with P-PCI to highlight its vascular network. The preparation of the air-filled mouse brain is described in Section 2.3.

### 2.2. CT Reconstruction

Generally, to determine the 3D structure of an object, multiple projections around the object are needed. In conventional X-ray CT, the 3D structure of the imaged object *f* is calculated with an FBP algorithm, expressed as

(6) $$\begin{array}{c} f\left(\;x,y,z\;\right)=\overset{\pi }{\underset{0}{\int }}\left(\;-\log\left(\;P_{\theta }\left(\;x,z\;\right)\;\right)\;\right)*h\left(\;x\;\right)d\theta , \end{array}$$

in which *P*_*θ*_ is the projection under projection angle *θ* and *h*(*x*) defines the 1D filter applied to the projections.

Conventional CT reconstruction is based on the Radon transform of the object, which is defined as the integration of absorption coefficients along a linear path. During the process of P-PCI, X-ray propagation direction is refracted at structural boundaries, which means the paths of X-rays cannot be assumed as lines. What is worse, in the measured phase-contrast images, both absorption-based and phase-based signals exist. Thus, reconstruction of a P-PCI CT with the FBP algorithm is mathematically incorrect. To address this problem, many phase-retrieval methods have been proposed and used in phase-contrast CT reconstruction [12]. In this paper, we adopt the Bronnikov-type algorithm in the image reconstruction, which combines the phase retrieval and FBP reconstruction together, named RP-FBP [13].

In our mouse brain imaging, the assumption of slowly varying absorption is reasonable. Thus, the absorption part inside the gradient operator can be taken out, in (5), yielding

(7) IDx,zI0x,z−λD2πI0x,z∇2φx,z=I0x,z1−λD2π∇2φx,z.

By observing the equation, the image formed in the detector plane consists of two parts: the absorption part and the X-ray phase shift with a second-order differential. With special consideration of the filter in the reconstruction, the pure absorption image *I*_0_(*x*) can be set to be equal to the incident X-ray intensity [10, 14]. Thus, the 3D structure of the sample can be reconstructed by

(8) $$\begin{array}{c} \delta \left(\;x,y,z\;\right)=\frac{1}{4\pi^{2}D}\overset{\pi }{\underset{0}{\int }}\left(\;1-I_{D}\left(\;x,z\;\right)\;\right)*q\left(\;x,z\;\right)d\theta \end{array}$$

with FT{*q*(*x*, *z*)} = *Q*(*ξ*, *η*) = |*ξ*|/(*ξ* ^2^ + *η* ^2^ + *α*), where FT{·} defines the Fourier transform operation, (*ξ*, *η*) corresponds to (*x*, *z*) in the Fourier space, and *α* is the compensation to the absorption of X-rays [10].

**Figure 2:**
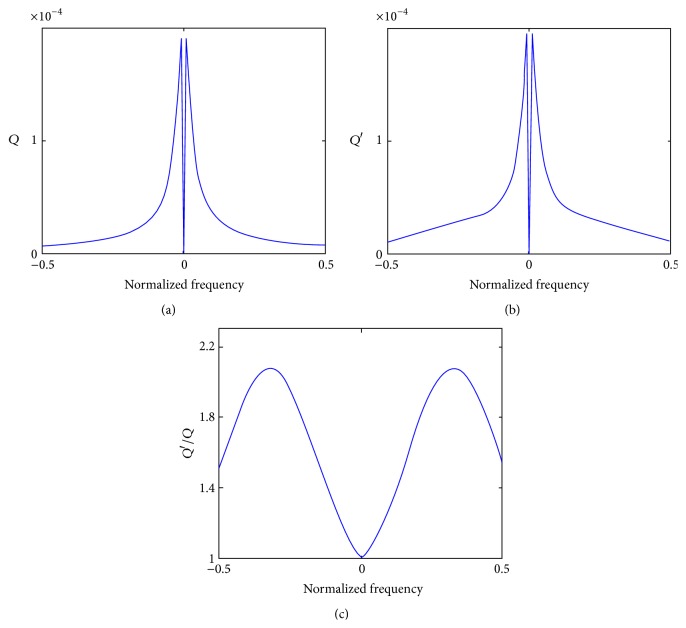
Reconstruction kernels used in phase-contrast tomography. Central lines of the two kernels are plotted in (a-b) and (c) shows their differences in normalized frequency domain.

As shown in Figure 2(a), the standard reconstruction kernel *q* in (8) is a low-pass filter in Fourier domain that has advantages in reduction high-frequency noise. Thus, the reconstructed image by (8) should have less noise and this phenomenon is also demonstrated by our experiment. The PR-FBP algorithm, as described in (8), is based on the ideal imaging condition that fully coherent X-ray beam illumination and projection are measured with a perfect detector. However, in practical P-PCI experiments, the X-ray source size is nonignorable and there is light diffusion in the detector system. The two aspects are major components of the point spread function (PSF) of the imaging system. Thus, (3) is expressed as

(9) $$\begin{array}{c} I_{D}\left(\;x,z\;\right)={\left|\;T\left(\;x,z\;\right)*P_{D}\left(\;x,z\;\right)\;\right|}^{2}*PSF\left(\;x,z\;\right). \end{array}$$

Usually, the system PSF is equivalent to a low-pass filter, which blurs the measured projection images. Taking the system PSF into the PR-FBP algorithm, (8) is rewritten as

(10) δx,y,z=14π2D·∫0π1−F−1FIDx,zFPSFx,z∗qx,zdθ.

In real imaging conditions, the system PSF is hard to apply to calibrate the imaging system since it will enhance the noise and degrade the imaging quality. So, to balance the image blurring and noise reduction, in our solution, the reconstruction kernel in (8) is redesigned to keep sample structures and prevent noise at the same time. The new designed kernel *Q*′ is plotted in Figure 2(b).  Figure 2(c) shows its difference with the standard reconstruction kernel *Q*.

### 2.3. Preparation of Air-Filled Mouse Brain and Hematoxylin and Eosin Staining

According to our previous study [8], the sudden density jump at air-tissue interface can introduce significant phase contrast for X-rays. Thus, in our* ex vivo* mouse brain imaging, we made an air-filled mouse brain whose vascular system was filled with air, making “air-tissue” interfaces.

The experiment followed the standard laboratory animal use procedure and experimental protocols were reviewed and approved by the Institutional Animal Care and Use Committee (IACUC) and the Bioethics Committee of School of Biomedical Engineering, Shanghai Jiao Tong University, Shanghai, China. Male adult CD-1 mouse (Sppir-BK Inc., Shanghai, China) weighing 30 grams was anesthetized with ketamine (100 mg/kg) and xylazine (10 mg/kg) intraperitoneally. Then, the chest and abdominal cavities were opened under an operating microscope (Leica, Wetzlar, Germany). 20 mL of heparinized saline was manually perfused transcardially, followed by 20 mL of 4% paraformaldehyde perfusion. Thereafter, the brain was taken out and placed in 4% paraformaldehyde for 24 hours at 4°C. The brain was further dehydrated at room temperature using graded ethanol. For imaging, the brain was air dried naturally after dehydration. For hematoxylin and eosin (H&E) staining, the brain was embedded in paraffin. Then, coronary paraffin sections with 4 *μ*m thickness of the brain tissue were prepared for H&E staining.

### 2.4. Imaging Experiments

The P-PCI experiment was performed at the BL13W beam line of Shanghai Synchrotron Radiation Facility (SSRF). A partially coherent X-ray beam is emitted from a wiggler source with energy of 16 keV. The sample was placed on a rotating stage that was 30 m away from the X-ray source. P-PCI projections of the sample were captured with a high spatial resolution detector (Photonic Science, Inc.) with pixel size of 7.4 × 7.4 *μ*m^2^. Coupled with a 2x microscope objective, the effective pixel size was 3.7 × 3.7 *μ*m^2^. The sample-to-detector distance was set at 0.6 m. In P-PCI CT scanning, 360 projection images were recorded over 180°. The exposure time for each image was 2 seconds.

To compare the performance of P-PCI, conventional X-ray imaging was also carried out. The prepared air-filled brain was imaged by a MicroXCT system (Xradia, Pleasanton, CA, USA). The voltage of the X-ray tube was set at 30 kVp and the current was 2 mA. The pixel size of detector is about 5 × 5 *μ*m^2^. The exposure time was 12 seconds.

## 3. Results and Discussion

### 3.1. Imaging Results of P-PCI

**Figure 3:**
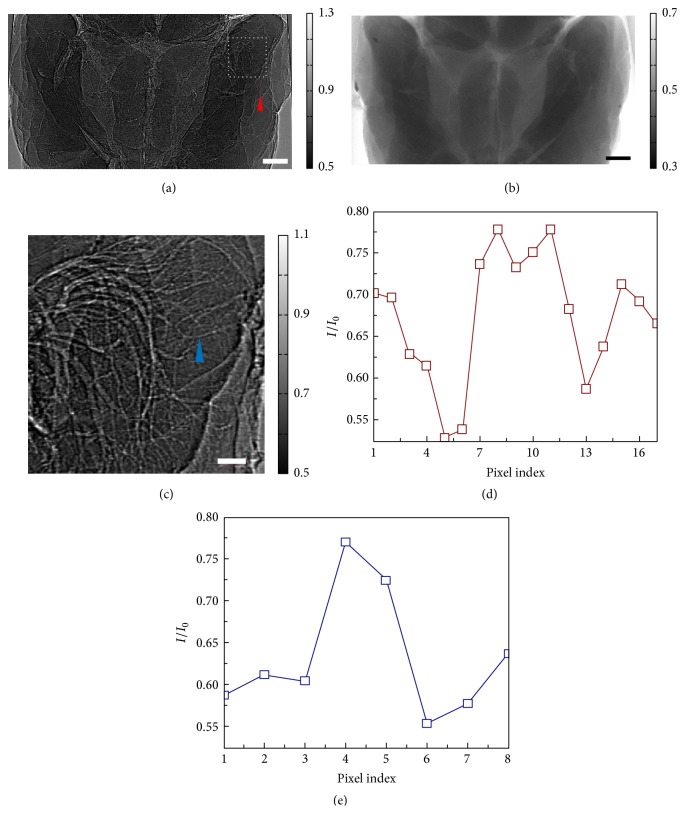
Comparison between P-PCI and conventional absorption-based X-ray imaging. The air-filled mouse brain was imaged by P-PCI method (a) and absorption-based X-ray imaging method (b), respectively, bar: 500 *μ*m. An enlarged view of the area outlined by the white dashed box in (a) is shown in (c), bar: 100 *μ*m. (d) and (e) plot intensity profiles of the cross-lines over blood vessels suggested by the blue and red arrows in (a) and (c).

The air-filled brain was prepared and imaged with both the P-PCI method and the conventional X-ray imaging method. Projections with different imaging modalities are shown in Figures 3(a)-3(b). Taking advantage of the edge-enhanced effect of the P-PCI method, tissue-air-tissue structured cerebral vessels are clearly observed in Figure 3(a).  Figure 3(d) plots the intensity profile of the cross-line over the vessel marked by the red arrow in Figure 3(a). The bright-dark fringe formed at the edges significantly improves its visibility.  Figure 3(c) is an enlarged view of the white box in Figure 3(a) in which there is an abundance of overlapped vessels. The cross-line of a small blood vessel, as indicated by the blue arrow, is plotted in Figure 3(e). For small diameter vessels, the tissue-air-tissue structure works like a convex lens bending X-rays from each side of the vessel and focusing them onto the single bright line along the vessel [8]. According to our results, with P-PCI method and air-filling preparation to the mouse brain, small blood vessels with a diameter of around 2 pixels can be effectively detected. On the contrary, patterns of blood vessels are absent in the image with conventional X-ray imaging method, as shown in Figure 3(b).

### 3.2. Numerical Simulation of P-PCI CT

The FBP reconstruction method can be directly applied to P-PCI CT data sets. Although there are exaggerated structure boundaries in reconstructed images, extra noise is also added. Here, we carried out a numerical experiment to study the noise of FBP reconstruction based P-PCI CT.

**Figure 4:**
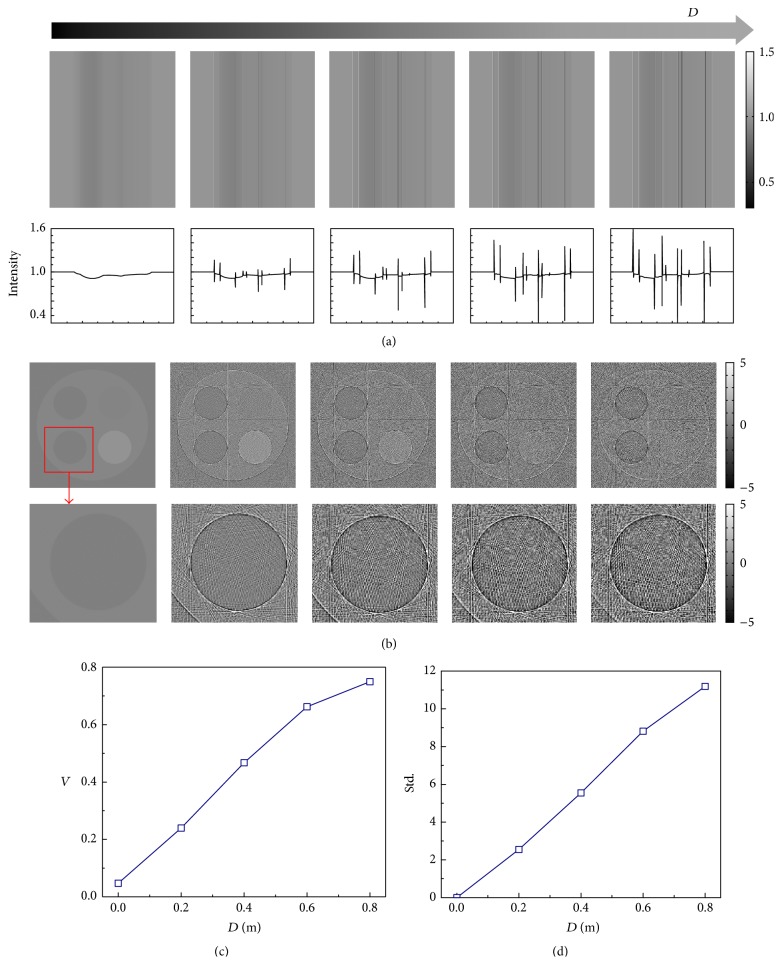
Simulation results of P-PCI CT. P-PCI projections of the numerical phantom and corresponding line profiles are listed in (a) along with the increasing of the sample-to-detector distance *D*. Their corresponding cross sections with direct FBP reconstruction and enlarged views of red-box areas are shown in (b). The visibility of each projection in (a) and standard deviation of the reconstructed slices from the basis image are plotted in (c) and (d), respectively.

In our numerical simulation, four materials, air, water, PMMA, and PTFE, were used to compose a phantom, as shown in Figure 4(b), and their absorption coefficients and refractive indices were obtained from the DABAX database [15]. The phantom was illuminated with fully coherent X-rays with energy of 25 keV. Projections were recorded by a detector with pixel size of 5 × 5 *μ*m^2^.  Figure 4(a) shows its projections with different sample-to-detector distance *D*, calculated using (3). Consistent with previous study [16], edges of the phantom are increasingly enhanced along with the distance *D*. The visibility of each projection image, defined as *V* = (*I*_max_ − *I*_min_)/(*I*_max_ + *I*_min_), is plotted in Figure 4(c). Here, *I*_max_ and *I*_min_ are the maximum and minimum intensities measured in the images. The P-PCI CT scan was performed with 180 projections taken at 1° increment. With direct FBP reconstruction, cross sections of the phantom are reconstructed and shown in Figure 4(b). As the increasing of edge-enhanced effect, the artifacts introduced by sharp edges in P-PCI projections become more and more significant as shown in the second row of Figure 4(b). Taking the reconstruction result of *D* = 0 as a basis, the standard deviations (Std) of each image are calculated and plotted in Figure 4(d). According to our simulation results, increasing sample-to-detector distance, enhancing edge visibility (Figure 3(c)), and introducing extra noise (Figure 3(d)) occur at the same time by applying the FBP reconstruction.

### 3.3. Reconstruction of Air-Filled Mouse Brain

**Figure 5:**
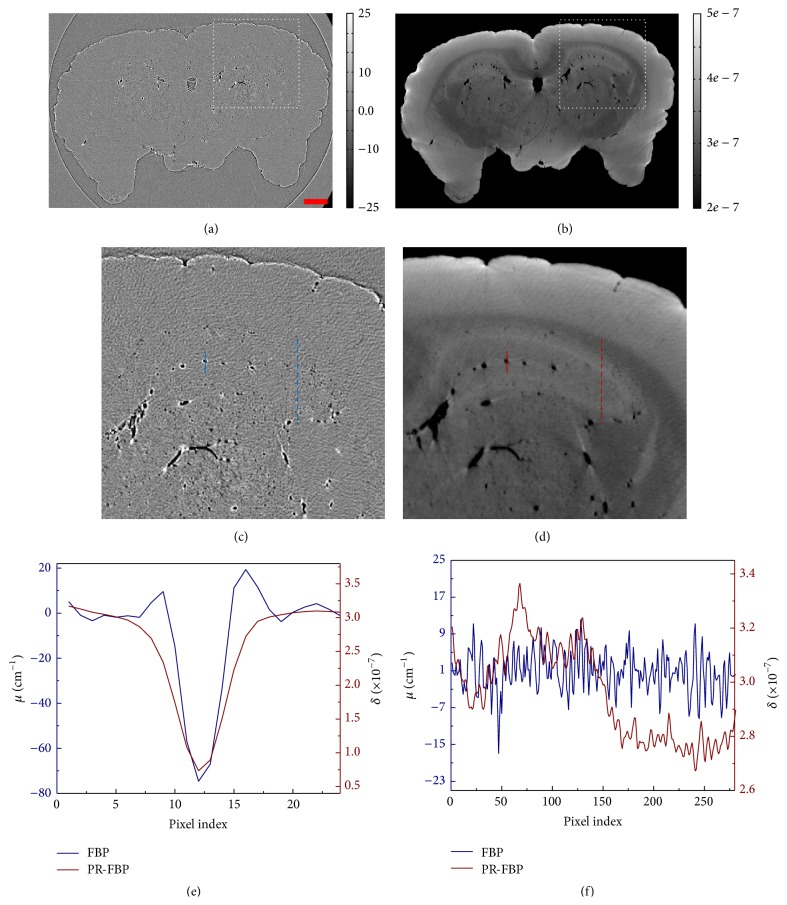
Reconstruction results of air-filled mouse brain with FBP and PR-FBP. The reconstructed images with FBP and PR-FBP methods are shown in (a) and (b), bar: 500 *μ*m. Enlarged views of the area outlined by white dashed boxes in (a) and (b) are shown in (c) and (d), accordingly. (e) plots cross-lines in (c) and (d) marked by solid lines and (f) plots cross-lines in (c) and (d) marked by dashed lines.

The P-PCI CT scanning of the air-filled mouse brain was performed at SSRF and the reconstruction results with FBP algorithm were shown in Figure 5(a). It is consistent with our simulation studies that there are obvious artifacts in the slice images as shown in Figures 5(a) and 5(c). The artifacts are radiating outward at air-tissue interfaces where the edge-enhanced effect occurs. Both numerical simulations and experimental results show that sharp edges in P-PCI projections introduce artifacts in the slice image by direct FBP reconstruction. Compared with the FBP reconstruction, the PR-FBP method retrieves the refractive index of the brain and then reconstructs the phase-contrast image instead. Without enhanced structural boundaries, the blood vessels are still observed with PR-FBP method and represented as black holes in slice images.  Figure 5(e) plots intensity profiles marked by solid lines in Figures 5(c)-5(d). Furthermore, as soft tissues are usually low-*Z* materials which are of low contrast in absorption-based X-ray images, the reconstructed image with PR-FBP method is superior to the soft-tissue imaging.  Figure 5(f) plots intensity profiles marked by dashed lines in Figures 5(c)-5(d). With the PR-FBP method, soft-tissue areas in the prepared mouse brain are observed in reconstructed slice images with PR-FBP algorithm which are all absent in Figure 5(c) with FBP algorithm.

**Figure 6:**
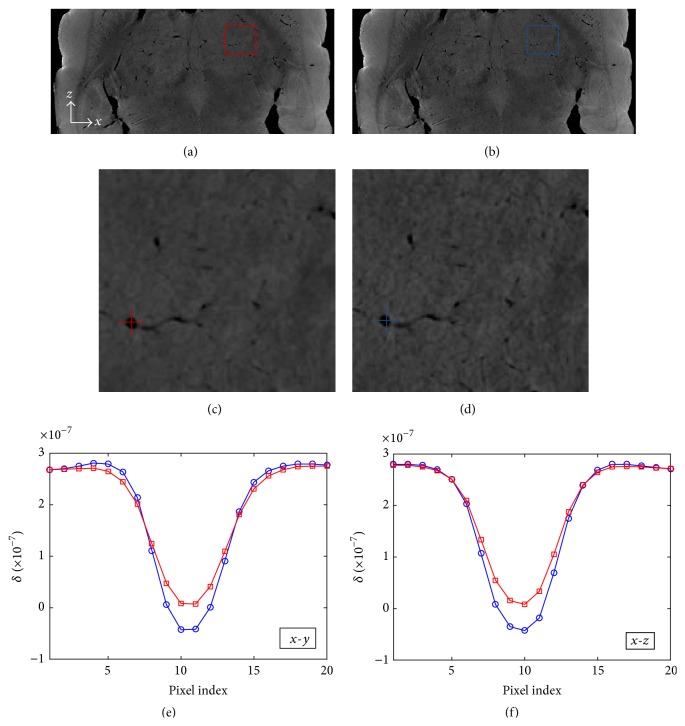
Comparison between results of PR-FBP (a) and PR-FBP with new designed kernel (b). (c) and (d) are their local enlarged images, as marked by red and blue boxes in (a) and (b), respectively. Line profiles in *x*-*y* plane and *x*-*z* planes are plotted in (e) and (f), corresponding in colors. *x*-*y* plane is the reconstructed plane; and *x*-*z* plane is extracted from stacked *x*-*y* planes.

As described in Section 2.2, due to the nonignorable X-ray source size and light diffusion in detector plane, the measured projection image is blurred by the system PSF. If the standard PR-FBP algorithm is used, the reconstructed sliced images should be oversmoothed. As shown in Figure 5(e), the red line, representing the line profiles over a blood vessel in Figure 5(d), suggests an oversmoothed vessel edge. To address this problem, we modified the reconstruction kernel in (8) and plotted the new designed kernel in Figure 2(b). The reconstruction result with new PR-FBP algorithm is shown in Figure 6(b) and compared with standard PR-FBP reconstruction. Figures 6(c)-6(d) are local enlarged views of red-box areas in Figures 6(a)–6(c). It is clear to see that the reconstructed image with new PR-FBP algorithm (Figure 6(d)) is much sharper than the conventional one as shown in Figure 6(c). Plotting line profiles over a blood vessel in both *x*-*y* plane and *x*-*z* plane, the results show that the new PR-FBP algorithm can well keep the shapes of blood vessels and has better image contrast which is beneficial to vessel segmentation applications. The reconstruction kernel in (8) can be further improved according to specific applications and imaging conditions.

### 3.4. Application

Both MRI and CT methods have limitations in imaging mouse brain, primarily the poor soft-tissue contrast of X-ray CT and poor spatial resolution of MRI. In this paper, we have proposed experiment scheme and reconstruction algorithm for the* ex vivo* study of mouse brain. The vascular system inside brain is filled with air and the whole brain is imaged with P-PCI. Since the high spatial resolution of P-PCI method and large phase shift introduced by air-tissue edges, the vascular network can be clearly revealed in two-dimensional projection and three-dimensional volume. For its kinds of functional areas, we employ RP-FBP algorithm and redesign the reconstruction kernel to keep structures and prevent noise. According to our experimental results, the vessels and soft-tissue areas in the imaged mouse brain are clearly represented with refractive indices, especially small cerebral vasculatures and low-contrast functional areas.

**Figure 7:**
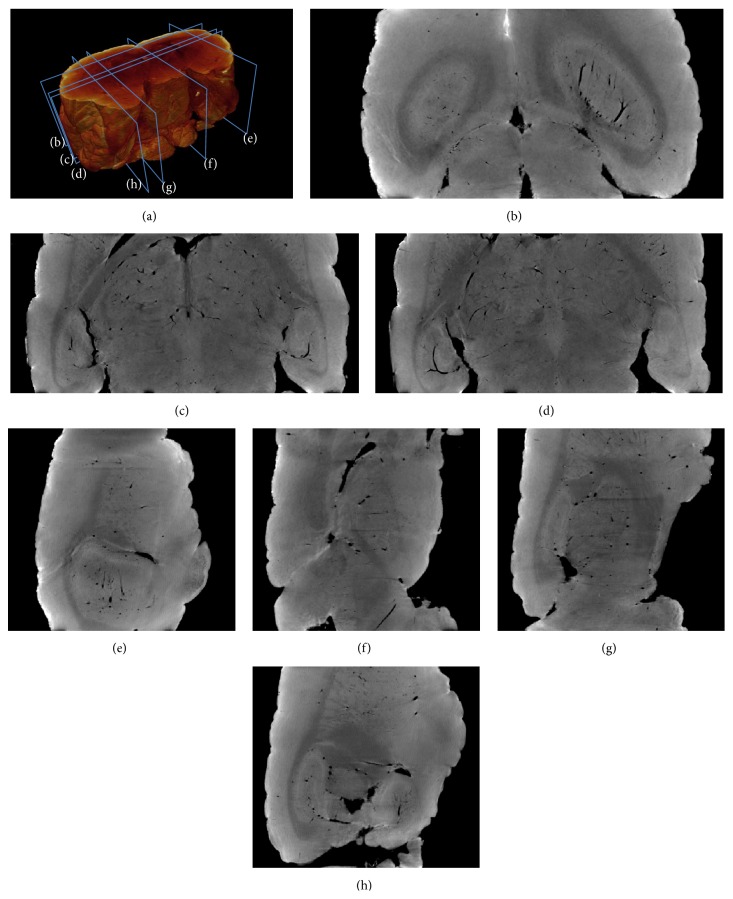
Coronal and sagittal sections of the reconstructed air-filled mouse brain. (a) is the three-dimensional rendering result of the air-filled mouse brain. (b–h) are cross-images corresponding to the labels in (a).

**Figure 8:**
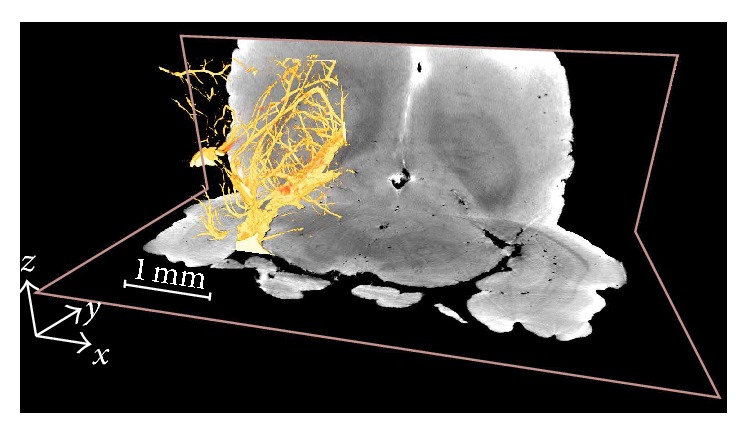
Three-dimensional visualization of party cerebral vasculature network.

By stacking phase-contrast slices together, we have the three-dimensional volume data of the imaged mouse brain. A rendering result of the air-filled mouse brain is shown in Figure 7(a). The coronal and sagittal sections of the brain are shown in Figures 7(b)–7(h) corresponding to labels in Figure 7(a). Functional structures are clearly represented in gray scale in Figures 7(b)–7(h). The three-dimensional visualization of party cerebral vasculature network is shown in Figure 8. According to the results in Figures 7 and 8, interesting areas can be positioned and segmented as the base for further studies. It should be noted that the images, in Figures 7 and 8, are reconstructed from raw P-PCI CT data, without additional preprocessing and postprocessing steps. Usually in a typical CT reconstruction, denoising methods are necessary in both projection domain and image domain for high-quality images [17]. So, in practice, the imaging quality of P-PCI CT can be further improved in specific imaging applications.

**Figure 9:**
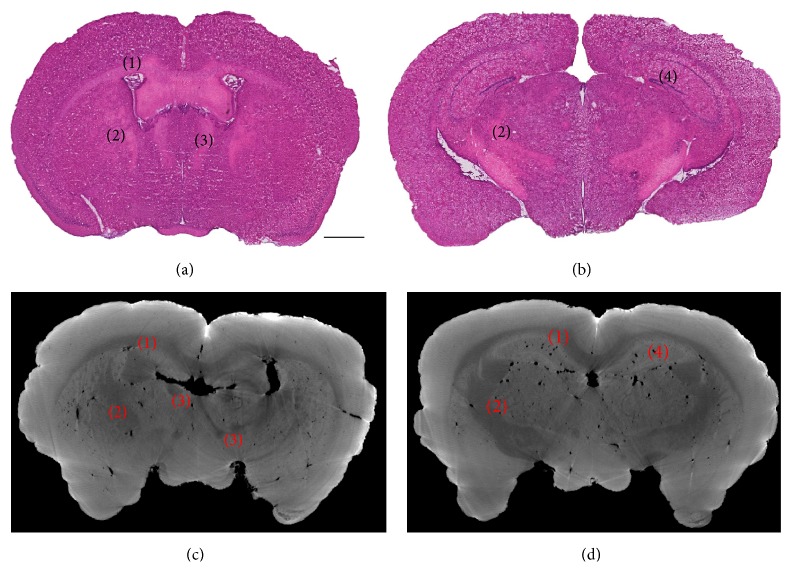
H&E-stained brain slices and reconstructed air-filled mouse brain images. (a) and (b) are two H&E-stained brain slices, bar: 1 mm. (c) and (d) are the reconstructed air-filled mouse brain images with almost the same position. In (c) and (d): (1) corpus callosum; (2) corpus striatum; (3) nucleus; (4) hippocampus.

In Figure 9, we compared the H&E-stained brain slices and the PR-FBP reconstructed images. It is well known that the H&E staining of tissue slices is a gold standard to study the tissue structure and cell arrangement in medical diagnosis. The H&E section image of mouse brain is shown in Figures 9(a)-9(b) in which corpus callosum, nucleus, and hippocampus structures are resolved with color. Correspondingly, in the reconstructed brain slices with the proposed method, these structures are also visible, as marked in Figures 9(c)-9(d). According to the result, the reconstructed brain slices can partially replace the functions of the H&E staining method and represent the internal functional structures in three-dimensional not two-dimensional slices.

## 4. Conclusion

The visualization of cerebral vasculature and functional areas are critical in the research of brain diseases. In this paper, we have presented experiment scheme and reconstruction algorithm for the 3D imaging of mouse brain using synchrotron radiation. An air-filled mouse brain was prepared and scanned using P-PCI CT. Taking both advantages of high spatial resolution of P-PCI and large phase contrast introduced by air-tissue boundaries, small cerebral vasculatures can be clearly resolved. Employing our redesigned PR-FBP algorithm, structure boundaries and undesired noise can be well balanced, and kinds of functional areas are presented which even partially replaced the standard H&E staining method. In conclusion, in this paper, we propose a sample preparation scheme and image reconstruction algorithm for* ex vivo* mouse brain imaging, offering a potential powerful imaging method for the research of brain disorders.
